# Symphyseotomy

**Published:** 1894-02

**Authors:** 


BUFFALO MEDICAL AND SURGICAL JOURNAL

A MONTHLY REVIEW OF MEDICINE AND SURGERY.

*EDITORS:*

THOMAS LOTHROP, M. D. - - WM. WARREN POTTER, M. D.

All communications, whether of a literary or business character, should be addressed to the managing editor: 284 FRANKLIN STREET, BUFFALO, N. Y.

VOL. XXXIII. FEBRUARY, 1894. No. 7.

### SYMPHYSEOTOMY.

THE revival within the past two years of this operation has marked an era in the obstetrical world. The operation was originally proposed in 1768 by Sigault, then a medical student in Paris. As is well known, it consists in a division of the symphysis pubis, with a view of allowing the pubic bones to separate sufficiently to admit of the passage of the child. According to Playfair, in 1778, the operation was performed thirteen times in Germany, France and Belgium; but once only in England, in 1782. It then gradually fell into disfavor, and finally became practically obsolete. In 1863, however, Morisani, of Naples, studied the operation on a dead subject, and three years later he operated on a living woman, saving both mother and child.

We have said that the operation has been revived within the past two years, for it is only within that time that it has come to be recognized in America as having a substantial basis. It is true, however, that it had been performed between January 1, 1886, and the close of 1892, in 115 cases in Europe and America, with a mortality of nine mothers and twenty-four children lost. Up to the close of 1892, it had been attempted but once, each, in England and Ireland. These figures are taken from Playfair, sixth American edition, edited by Harris, and may be regarded as substantially correct, for Dr. Harris seldom errs in his statistics.

During the year 1893, almost every obstetrical surgeon in the larger cities of America has performed symphyseotomy one or more times, and the record of successful work in this field is of a most encouraging nature. This operation, however, can never take the place of the Cesarean section in extreme cases of pelvic deformity, but it may be properly offered as a substitute for craniotomy, in those slighter cases that are just too small to admit the passage of a living child. Prof. J. Edwin Michael, of Baltimore, has performed the operation successfully where there was impaction in consequence of mal-presentation—a case in which craniotomy would formerly have been considered necessary. The two operations, symphyseotomy and Cesarean section (Sänger and Porro), ought to drive craniotomy from the field, and make it no longer considered as an alternative.

Symphyseotomy is less difficult than Cesarean section or Porro's operation, or even than a difficult craniotomy, though Chroback, of Vienna, thinks that it is not less dangerous than Cesarean section, and demands a more complicated apparatus.

The method of performing the operation is as follows: the symphysary cartilage is incised with a blunt-curved knife, or sawed with a straight or chain saw, from above downward. After the incision, there is generally much hemorrhage, which is stopped with a plug of iodoform gauze, supplemented by counter pressure from the vagina. After the passage of the child, sutures should be introduced, uniting the pubic bones. Some surgeons have contended that sutures were unnecessary, but we think it safer to apply them. After this, the legs are extended and brought back, and the pelvis is surrounded by a large band of plaster, and shored up with pillows or bran bags.

With the expectant method, women may be delivered without operation who have eight centimeters of useful diameter; version permits the extraction of children through a diameter of seven and five-tenths centimeters, while the lowest limit for a symphyseotomy would be from six to six and three-tenths centimeters. The operation is to be avoided among primiparæ, and it should be practised only when the introitus is completely dilated, else it may cause grave lesions to the vagina. It is not an operation to be practised by the inexperienced, but should be left in the hands of the most competent obstetric surgeons. All the details of aseptic surgery should carefully be followed, and every safeguard known to modern surgery employed.

---

THE *Medical Mirror*, St. Louis, for December, 1893, publishes an original lecture on Intestinal Indigestion, by Thomas Hunt Stucky, M. D., Professor of the Theory and Practice of Medicine in the Hospital College of Medicine, Louisville, Ky., delivered by invitation of the faculty of Marion-Sims College of Medicine to the class and invited members of the medical profession, Tuesday evening, November 21, 1893. This is an able and exhaustive exposition of the subject, and all interested should procure the December number of the *Mirror*.

